# Sleep Traits, Night Shift Work and Lung Cancer Risk among Women: Results from a Population-Based Case-Control Study in France (The WELCA Study)

**DOI:** 10.3390/ijerph192316246

**Published:** 2022-12-04

**Authors:** Emilie Cordina-Duverger, Shreeshti Uchai, Nastassia Tvardik, Régine Billmann, Diane Martin, Jean Trédaniel, Marie Wislez, Hélène Blons, Pierre Laurent-Puig, Martine Antoine, Pascal Guénel, Loredana Radoï, WELCA Study Group

**Affiliations:** 1 Équipe Exposome et Hérédité, Inserm U 1018, Center for Research in Epidemiology and Population Health (CESP), University Paris-Sud, Université Versailles Saint-Quentin-en-Yvelines, Université Paris-Saclay, 94807 Villejuif, France; 2 École des Hautes Etudes en Santé Publique (EHESP), 93210 Paris, France; 3 Unité INSERM UMR-S 1124, Toxicologie, Pharmacologie et Signalisation Cellulaire, Groupe Hospitalier Paris Saint Joseph, Université de Paris, 75014 Paris, France; 4 Unité d’Oncologie Thoracique, Institut du Cancer Paris Carpem, Assistance Publique Hôpitaux de Paris (AP-HP Centre), Université Paris Cité, 75014 Paris, France; 5 Centre de Recherche des Cordeliers, UMRS 1138 Complement, Inflammation and Cancer, Université de Paris, 75006 Paris, France; 6 Department of Biology Physiology and Genetics, Institut du Cancer Paris Carpem, Assistance Publique Hôpitaux de Paris (AP-HP Centre), Université Paris Cité, 75005 Paris, France; 7 Centre de Recherche des Cordeliers, INSERM, CNRS SNC 5096, Sorbonne Université, Université de Paris, 75006 Paris, France; 8 AP-HP, Tenon Hospital, Pathology, 4 Rue de la Chine, 75020 Paris, France; 9 UPMC Université Paris 06, GRC No. 04, Theranoscan, 75020 Paris, France; 10 UFR d’Odontologie, Assistance Publique Hôpitaux de Paris (AP-HP Nord), Hôpital Louis Mourier, Université Paris Cité, 92700 Paris, France

**Keywords:** lung cancer, women, night shift work, sleep disorders, sleep duration, chronotype

## Abstract

Circadian rhythm disruption due to night shift work and/or sleep disorders is associated with negative health outcomes including cancer. There is only scant evidence of an association with lung cancer, unlike breast and prostate cancer. We explore the role of sleep disorders and night shift work in lung cancer risk among women in a population-based case-control study, including 716 lung cancer cases and 758 controls. Multivariable logistic regression models were used to estimate odds ratios (OR) and 95% confidence intervals (CI) associated with sleep duration per day (<7 h, 7–7.9 h, ≥8 h), a summary index of sleep disorders, chronotype, and night shift work exposure metrics. When compared to women with an average sleep duration of 7–7.9 h per day, the OR was 1.39 (95% CI 1.04–1.86) in long sleepers (≥8 h) and 1.16 (95% CI 0.86–1.56) in short sleepers (<7 h). Overall, lung cancer was not associated with the sleep disorder index, nor with night shift work, regardless of the duration of night work or the frequency of night shifts. However, elevated OR associated with the sleep disorder index were found in the subgroup of current smokers. The U-shaped association of lung cancer with sleep duration was more particularly pronounced among women who worked at night ≥5 years. Our findings suggested that sleep patterns are associated with lung cancer risk in women with a potential modifying effect by night shift work duration or tobacco smoking.

## 1. Introduction

Lung cancer is the leading cause of cancer incidence and mortality worldwide. With an estimated 2.2 million new cases and 1.8 million deaths in 2020, it accounted for 11.4% of the total new cancer cases and 18.0% of the total cancer-related deaths [1].

Tobacco smoking is by far the major risk factor for lung cancer, but the etiology of lung cancer is not fully understood, particularly regarding other environmental or hormonal risk factors in women [2].

Night shift work as well as sleep disorders leading to a misalignment of the biological clocks with the day-night cycle have been suggested as possible causes of health disorders including cancer [3]. Artificial illumination at night suppresses human melatonin secretion and disrupts circadian rhythmicity [3]. In addition to its central role in the regulation of sleep and wake cycles, melatonin is involved in the modulation of the immune system, tumor growth inhibition and anti-aging processes. Several mechanisms have been proposed to link sleep disturbances and circadian rhythm disruption with cancer development and growth, including melatonin physiopathology, immune suppression, and cancer-stimulatory cytokines activation [4,5].

Sleep is a multidimensional concept characterized by quantitative and qualitative parameters. Sleep duration has been inconsistently associated with risk of various cancers [6,7,8,9,10,11,12]. Long sleep duration has been associated with risk of prostate cancer [13], breast cancer [6,14], colorectal adenoma [7], and hematological malignancies [15,16], while other studies did not report any association of sleep duration with prostate [17,18] or breast cancer [19].

The relationship between sleep duration and lung cancer risk has been studied in a few studies that reported an increased risk in short sleepers as well as in long sleepers as compared to normal sleepers (7–8 h per day) [20,21,22]. However, this U-shaped relationship was not confirmed in other studies [16,23,24] and a meta-analysis [25]. Beside sleep duration, Xie et al. [22] have examined the association of lung cancer with insomnia or snoring but found no association, while a study in the UK Biobank cohort [26] concluded that the effects of long sleep duration, frequent insomnia symptoms and evening chronotype may combine to increase lung cancer risk. Finally, sleeplessness was associated with lung cancer in a Mendelian randomization analysis [27].

Sleep disorders may be a mediator of the association between night work and cancer. Night shift work was classified by IARC as a probable carcinogen (group 2A) in its 2019 monograph based on convincing evidence from studies on breast and prostate cancers while the evidence of an association with lung cancer was considered inadequate due to the limited number of studies available [28]. In the Nurses’ Health study, women with ≥15 years of rotating night shift work had modest increase in lung cancer incidence [29] and mortality [30]. In a Canadian case-control study, an increased risk of lung cancer in men who ever worked at night was found [31]. Conversely, no association with night shift work was observed in female textile workers in China [32] nor in male chemical workers in Germany [33]. In contrast to these studies, a recent meta-analysis reported a decrease in the risk of lung cancer in night shift workers [34].

Regular smoking impairs nightly sleep structure. Smokers had shorter sleep duration, increased difficulty falling asleep or maintaining sleep [35] due to the effects of the nicotine on the central nervous system [36]. Although all epidemiological studies analyzing the association between lung cancer and sleep patterns have taken into account tobacco smoking [20,21,22,23,24,26,27], few of them have investigated the modifying effect of smoking status on the association between sleep characteristics and lung cancer by conducting stratified analyses or by testing for interaction [20,24,26]. Khwaja et al. suggested no effect of smoking status [24], while Luojos et al. [20] and Peeri et al. [26] found a stronger U-shaped association for both short and long sleep durations among current smokers.

In this paper, we analyzed the risk of lung cancer in relation to night shift work and sleep traits, including sleep duration, sleep disturbances and chronotype, using data of a case-control study on lung cancer in women conducted in the greater Paris area. Since tobacco smoking and night shift work can enhance sleep disturbances, we also examined their effects on the association between sleep traits and lung cancer.

## 2. Materials and Methods

### 2.1. Study Population

The WELCA study (Women Epidemiology Lung Cancer) was described in details elsewhere [37]. The WELCA study is a multicenter population-based case-control study conducted between 2014 and 2017 among women 18 to 75 years old living in the Ile-de-France region, which comprises 8 “départements” (administrative areas).

Cases were women diagnosed with incident, histologically confirmed, primary lung cancer [International Classification of Diseases (ICD) 10th revision codes C34] [38] recruited in pneumology and oncology departments of public hospitals. Lung cancer of all histological types, excluding carcinoid tumors, were included in the study. From 849 eligible cases identified in the participating centers, 47 refused or were too ill to participate, 28 died before the interview, 27 could not be contacted, and 31 had missing information on night shift work history and sleep patterns leaving 716 cases available for the analysis (participation 84.3%).

Control women were recruited in the general population of Île-de-France, using random selection of phone numbers in the telephone directory. Controls were frequency-matched to the cases by 5-year age group and “département”. In order to minimize selection bias that may arise from differential response rate across categories of socio-economic status (SES), quotas were applied to the control group to reflect the distribution by SES of women of the same age in the general population. From 1107 eligible controls contacted by phone, 256 refused to participate, 67 could not be reached, and 22 were too ill to participate. In addition, 4 controls who responded to the summary questionnaire were excluded. In total, 758 controls were available for the present study (participation 68.3%).

Each subject signed an informed consent. The ethical approval for the WELCA study was obtained from the Institutional Review Board of the French National Institute of Health and Medical Research and by the French data Protection Authority (IRB-Inserm, no. 3888 and CNIL no. C13–52).

### 2.2. Data Collection

In-person interviews of cases and controls were conducted by trained research nurses using a standardized questionnaire. We collected information on socio-demographic characteristics, reproductive and hormonal history, personal and family medical history, lifestyle-related factors (tobacco smoking, alcohol drinking, anthropometric characteristics and recreational physical activity) and lifetime occupational history. The date of diagnosis of the cases and the date of interview of the controls was used as a reference date. Only exposures that occurred before reference date were considered in the analysis.

#### 2.2.1. Sleep Traits

The sleep history of each participant was documented from the age of 20 until the date of reference. Subjects were asked to report sleep traits including sleep duration (in hours) and five sleep-related disturbances (no/yes/unknown): difficulty in falling asleep during several months, waking up too early, waking up too tired, waking up at night, and intake of sleep medicine. For each period of significant change of sleep traits, a date of start and end was declared.

The average sleep duration over lifetime was calculated as the time-weighted average of the sleep duration reported by the participant in the different periods declared and categorized as <6 h, 6–6.9 h, 7–7.9 h, 8–8.9 h and ≥9 h. The categories were subsequently grouped (<7 h, 7–7.9 h and ≥8 h) in the stratified and polytomous analyses to have a sufficient number of subjects in each category for each variable. Further, we created a sleep disorder index (SDI) by adding up the number of sleep-related disturbances reported by the participants. Each disturbance (difficulty in falling asleep, waking up too early, waking up too tired, waking up at night, and intake of sleep medicine) reported at least once in the lifetime counted for 1. The SDI ranged from 0 to 5 and was categorized in 3 categories: low (0 or 1 disturbance), medium (2 or 3 disturbances) or high (4 or 5 disturbances).

To assess the chronotype, women were asked if they were rather a morning or evening or neither morning nor evening person.

#### 2.2.2. Night Shift Work Exposure

For each job held for at least six months during lifetime, subjects were asked if they worked at least one hour between 00:00 a.m. and 05:00 a.m., the working time schedules, and the frequency of night shift work (never, occasionally, often, always). Each subject reported dates of start and end for each night job.

Women were classified as night shift workers if they had ever worked for at least 3 h between 00:00 a.m. and 05:00 a.m. [39]. Night workers were further characterized by duration of night shift work (<3 years, 3–4.9 years, 5–9.9 years and ≥10 years) and by frequency of night shift work (occasionally, often, always) based on the job with the highest frequency during work history. Because few women had long nigh work duration, in the stratified and polytomous analyses we used the median of night work duration among controls as cut-off to categorize this variable (<5 years, ≥5 years). For all analyses, “never night shift work” was used as the reference category.

#### 2.2.3. Tobacco Smoking

Information on the tobacco smoking history was obtained for each smoking period using years of start and end and the number of cigarettes smoked per day. Smoking status was categorized as never smokers (women who smoked less than 100 cigarettes over the lifetime), former smokers (women who quit smoking for at least 2 years before the reference date) and current smokers. The lifetime smoking history was modelled using the Comprehensive Smoking Index (CSI), an aggregate parsimonious score that accounts for the most important smoking metrics (intensity, duration and time since quitting) [40].

### 2.3. Statistical Analysis

Odds ratios (ORs) and 95% confidence intervals (95% CI) of lung cancer associated with sleep traits and night shift work metrics were calculated from logistic regression models. All models were adjusted for the frequency-matching variables, i.e., age (<50 years, 50–59 years, 60–69 years and ≥70 years) and area of residence (8 “départements” of the Ile-de-France region, see Table 1), as well as all relevant covariates associated with the lung cancer: CSI (continuous), marital status (never lived with a partner; living with a partner; separated or widowed), socio-economic status (executives and higher intellectual professions, intermediate occupations, employees, and others), and BMI 2 years prior to interview (continuous).

The association of lung cancer risk with sleep traits was also studied in stratification analyses by smoking status, night shift work and chronotype. Interactions test between sleep traits and stratification variables were performed using Wald test.

Polytomous (multinomial) logistic regression was used to estimate the ORs and 95% CI for lung cancer associated with sleep traits, chronotype and night shift work by histological subtypes. The ORs for the different histological subtypes were compared using the OR homogeneity test.

The statistical analyses were performed using SAS (Statistical Analysis Software 9.4, SAS Institute Inc, Cary, NC, USA).

## 3. Results

### 3.1. Selected Characteristics of the Study Population

The cases and controls were similarly distributed in terms of age and area of residence (Table 1); 51% of cases and 43% of controls were married or living with a partner. Compared to controls, cases were less often employees (37% vs 41%), held intermediate occupations less frequently (33% vs 37%), and had more often executive and higher intellectual professions (21% vs 16%) (*p* < 0.001). Cases and controls differed in terms of tobacco smoking (52% of cases were current smokers vs 23% of controls; 53% of cases were in the highest two quartiles of CSI vs. 16% of controls) (*p* < 0.0001). Overall, cases had a lower BMI 2 years before the interview than controls (65% of cases had underweight or normal weight vs 51% of controls) (*p* < 0.0001).

Among 716 incident lung cancer cases, the majority of the patients had adenocarcinoma (72%) followed by small cell carcinoma (12%) and squamous-cell carcinoma (9%).

### 3.2. Sleep Traits, Night Shift Work and Lung Cancer

A U-shaped relationship between sleep duration and lung cancer was observed (Table 2). When compared to normal sleepers (7–7.9 h/day), the ORs for long sleepers (≥ 8 h) and short sleepers (<7 h) were 1.39 (95% CI: 1.04–1.86) and 1.16 (95% CI: 0.86–1.56), respectively. The ORs associated with the SDI were close to unity regardless of the score value. When compared to neutral chronotype, the ORs for the morning and evening chronotypes were slightly increased and close to statistical significance for morning chronotype (OR 1.38; 95% CI: 0.96–1.99). Twelve percent of cases and controls had ever worked during night shifts. Lung cancer risk was not associated with previous night work nor with duration or frequency of night work.

Table 3 shows that the U-shaped association of sleep duration with lung cancer did not change substantially according to smoking status, with increased ORs for long sleepers among never smokers (OR 1.40, 95% CI: 0.84–2.32), former smokers (OR 1.33, 95% CI: 0.78–2.27) and current smokers (OR 1.48, 95% CI: 0.87–2.52). In current smokers, the ORs for medium and high SDI were increased, and reached statistical significance for the latter (OR 1.88, 95% CI: 1.09–3.26), while they were decreased in never and former smokers (*p* interaction between SDI and smoking status = 0.03). As compared to neutral chronotype, the ORs for morning and evening chronotypes were both increased in never and current smokers, while they remained close to unity in former smokers. The ORs for night shift work were not increased in never smokers, but were slightly elevated in former and current smokers (*p* interaction = 0.11). The ORs did not increase linearly with duration of night shift work in either group of smokers.

Table 4 shows the associations between sleep duration, SDI and chronotype and lung cancer risk according to night work. The U-shaped relationship with sleep duration was particularly marked in women who worked at night for 5 or more years with increased OR in both short sleepers (OR 3.23, 95% CI: 1.05–9.90) or long sleepers (OR 1.78, 95% CI: 0.53–5.99). There was also indication of an association between lung cancer risk and high SDI in night shift workers ≥5 years (OR 2.75, 95% CI: 0.84–9.02). Changing the cut-off of the duration of night work to 10 years showed similar results, with increased estimates which were less precise due to the small number of subjects in the stratum corresponding to the longest duration of night work (Supplementary Materials, Table S1).

The ORs for each histological subtype of lung cancer associated with sleep and night work variables are shown in Table 5. No associations between lung cancer and sleep traits, chronotype or night shift work variables were found. The U-shaped association of the ORs with sleep duration was observed for adenocarcinoma (OR 1.27, 95% CI: 0.93–1.74 in short sleepers and OR 1.43, 95% CI: 1.05–1.94 in long sleepers, in comparison with normal sleepers). Results for other histological subtypes were based on small numbers and were not significant, except for small cell carcinoma in women with morning chronotype (OR 2.64, 95% CI: 1.01–6.91). Paired comparisons between histologic subtypes were not statistically significant regardless of the sleep or night work variables. Analyses restricted to non-smoking cases diagnosed with adenocarcinoma and non-smoking controls showed similar results to those observed in non-smokers overall or in all adenocarcinoma cases vs. all controls (data not shown).

Associations between lung cancer and sleep duration, SDI and night shift work according to chronotype were analyzed (Supplementary Materials, Table S2). We did not observe any differences in lung cancer risk according to the three chronotypes.

## 4. Discussion

This study provides new insights into the relationship between sleep duration, sleep disorders, chronotype and night shift work and lung cancer in women. We found that short sleepers (<7 h/day) and long-sleepers (≥8 h per day) were at an increased risk of lung cancer as compared to normal sleepers. A more pronounced association with lung cancer was suggested in short sleepers who worked at night for 5 or more years, as well as in current smoking women with high SDI. Our results suggested that smoking status or duration of night shift work, could modify the association between sleep disorders and lung cancer incidence.

### 4.1. Sleep and Lung Cancer

We observed a U-shaped relationship between sleep duration and lung cancer risk though only women who slept ≥8 h had a 1.4-fold significantly increased risk of lung cancer when compared to those who slept 7 h. Women having a sleep duration shorter than 7 h had a 1.2-fold non-significantly elevated risk of lung cancer. This is similar to the results from a cohort study conducted among farmers in China, which reported a J-shaped relationship between sleep duration and lung cancer mortality [21], with increased risks among both men and women whose sleeping hours were either greater or less than 8 h per day. A prospective population-based cohort from Eastern Finland in men reported similar findings, where sleep durations of less or more than 7–7.5 h were associated with increased lung cancer risk (U-shaped relationship), with even stronger associations among current smokers [20]. In the UK Biobank cohort study, a U-shaped association was observed between sleep duration and lung cancer risk, with an 18% higher risk for short sleepers (<7 h) and a 17% higher risk for long sleepers (>8 h) compared with normal sleepers (7–8 h) [22]. An updated analysis in the same cohort showed that long sleepers (>8 h) had a 21% increased lung cancer risk compared with normal sleepers (7–8 h) [26]. Contrary to these studies, two prospective cohort studies conducted among female Californian teachers [23] and US male physicians [24] reported no association between lung cancer risk and sleep duration.

Sleep is a multidimensional concept, including chronotype, sleep duration, sleep deprivation, getting up in the morning, daytime napping, and insomnia. Therefore, sleep duration may not sufficiently account for quality of sleep [26]. Sleep disruption resulting from sleep deprivation, disturbances or restricted sleep is a potential risk factor for cancer [11,12]. A Mendelian randomization study based on data from UK Biobank and International Lung Cancer Consortium showed an increased risk of lung cancer (overall and adenocarcinoma) associated with sleeplessness and an inverse association between lung cancer risk (overall but not adenocarcinoma) and sleep duration [27]. Two others studies based on the UK Biobank cohort found inconsistent results: insomnia and snoring were not associated with lung cancer incidence in the study of Xie et al. [22], while usually insomnia symptoms increased the risk of lung cancer compared with never/rarely experiencing symptoms in the study of Peeri et al. [26]. In our study, no overall association between the risk of lung cancer and several sleep disorders (i.e., difficulty in falling asleep, waking up too early, waking up too tired, waking up at night, and intake of sleep medicine) was detected. However, higher risk of lung cancer was found in women with both high SDI and tobacco smoking or high SDI and long duration of night work.

In two studies, subjects with evening chronotype were at higher risk of lung cancer compared with those with morning chronotype [22,26]. In our study, there was no significant difference in the risk of lung cancer between women with morning or evening chronotype, although the OR was slightly higher in those with morning chronotype.

Smoking may impact sleep duration and quality. Indeed, current smokers had poorer sleep than never smokers (less total sleep time, longer latency to sleep onset, increased difficulty falling asleep, maintaining sleep, and waking up earlier than desired) [35]. Regular smoking impairs the nightly sleep structure due to the biological effects of nicotine on the central nervous system; nicotine stimulates the release of aminergic neurotransmitters (e.g., dopamine and serotonin) and thus disturb normal regulation of sleep toward lighter stages of sleep [36]. The results of the few epidemiological studies on the relationship between sleep traits and lung cancer by smoking status are inconsistent. Khwaja et al. suggested no effect of smoking status on the association between sleep duration and incidence of lung cancer [24], while Luojos et al. [20] and Peeri et al. [26] found a stronger U-shaped association for short and long duration of sleep among current smokers. In our study, we did not find an effect of smoking status on the association between sleep duration and lung cancer. However, the risk of lung cancer (after adjustment for CSI) was observed to be 2-fold higher among current smoking women with high SDI when compared to those with low SDI.

The biologic mechanisms underlying the potential association between sleep disorders and incidence of cancer are complex. Sleep deprivation could result in immune suppression, activate cancer-stimulatory cytokines and finally promotes cancer [4,5]. In contrast, long sleep duration could indicate ill-health, which could in turn increase cancer incidence [10]. Indeed, poor general health, with depressive symptoms, low socio-economic status or high BMI, have been shown to be related with long duration of sleep [41,42,43]. In addition, two recent meta-analyses have shown that sleep-disordered breathing is an independent risk factor of lung cancer [44,45].

### 4.2. Night Shift Work

Overall, lung cancer risk in our data was not associated with night shift work duration or frequency. These findings are consistent with a few previous studies. No association between lung cancer and long term rotating night shift work was observed in a cohort study conducted among female textile workers in China [32], in a cohort study in male and female rotating or night shift workers in Sweden [46], and in a cohort of male shift workers [33]. Similarly, a population-based cohort study investigating cancer risk associated with various occupations suggested no increased risk of lung cancer among male and female shift workers [47]. A French population-based case-control study did not identify any occupations requiring shift work to be associated with the lung cancer risk among men and women [48]. In our study, the most frequent occupations among night shift workers were healthcare workers and travel crews. However, due to the small numbers in our study population we were unable to carry out analyses limited to these groups.

Contrary to studies cited previously, exposure to night shift work for 15 years or longer was reported to be associated with an increased lung cancer incidence [29] and mortality among female nurses in the USA [30]. In our study, night shift work durations were short, only 2% of cases and 3% of controls having done night shift work for 15 years and more. A Canadian population-based case-control study found an elevated risk of lung cancer among males who had ever worked at night compared to those who had never been night workers, but there was no evidence of increasing risk with increasing duration of night work. The risk was comparable between different histological subtypes [31]. Similarly, in our study, no difference was found between different histological types of lung cancer with respect to the exposure to night shift work.

### 4.3. Combined Effect of Sleep Traits and Night Shift Work

Sleep traits and night shift work may lead to disruption of circadian rhythms. Night shift work is commonly associated with disturbed sleep [49]. They could act synergistically to increase the risk of lung cancer. Although we found no overall association between lung cancer and night shift work exposure metrics, our data suggest that lung cancer risk is increased among short sleepers who reported working at night for ≥5 years. Similarly, women with high SDI (cumulating in 4 or 5 sleep disturbances) who did night work for ≥5 years were at an increased risk of lung cancer, again suggesting a combined effect of both factors. However, these results are based on small numbers and require confirmation by larger studies.

The mechanism by which night shift work may increase lung cancer risk involves the disruption of melatonin secretion. It has been shown that external environmental factors, such as unnatural light at night, alter melatonin secretion [50], which have a naturally occurring peak between 02:00 a.m. and 04:00 a.m. [51]. There is consistent evidence from both animal and in vitro models indicating that melatonin may have anti-carcinogenic effects, such as anti-oxidant, anti-apoptosis, and anti-angiogenesis, as well as modulation of hormones and immunity [52], including on lung carcinogenesis [53]. In addition, tumor suppression is a clock-controlled process. Night-shift workers are exposed to dysfunction of circadian genes that is understood to play a role in DNA repair and carcinogen metabolism. The disruption of the circadian rhythm is associated with negative health outcomes including cancer initiation and growth [3].

### 4.4. Strengths and Limitations of the Study

The main strengths of this study focusing on lung cancer in women include the large sample size and the availability of detailed information about different night work and sleep variables throughout life for each subject. Cases were recruited in Paris pneumology and oncology departments of public hospitals. Implementing the study in a densely populated area enabled including almost all the clinical wards that treat lung cancer patients and optimizing the number of eligible female lung cancer patients. The participation rates were high in cases and controls, but selection bias could not be ruled out entirely. This study was retrospective in design, making it prone to recall bias. However potential recall bias was minimized using a standardized questionnaire administered by trained re-search nurses, resulting in increased quality of data. Face to face interviews were conducted shortly after diagnosis of cases (70 days in average) in or-der to curtail the risk of survival bias. Further, to reduce the risk of selection bias while selecting the controls, controls were randomly selected from the general population residing in the study area following an incident density sampling with the help of a polling institute with extensive experience in this area. For recruiting the controls, we applied quotas by socioeconomic status (SES) to minimize selection bias that may arise from differential participation rates by SES category. The probability of healthy worker bias among night shift workers cannot be excluded as usually the jobs requiring night shift work might require a healthier physical profile, subsequently leading to no or decreased risk for lung cancer among the night shift workers.

Our study was comprised of 12% of night shift workers, which is consistent with the proportion observed among French women in 2015, i.e., 9.9% [54]. However, while carrying out stratified analysis, categories of night work and sleep indicators had low frequencies, leading to a lack of power for detecting associations.

Detailed information on a wide range of socio-demographic and lifestyle related factors, including smoking habits, was available in this study, thus enabling to account for their potential confounding effect. However, despite careful consideration of tobacco smoking through the CSI, residual confounding—resulting in an overestimation of risk in smokers—cannot be completely ruled out. In addition, information on other risk factors, such as occupational exposures, indoor or outdoor pollution or diet was not available.

We attempted to explore different domains of sleep, such as sleep duration, chronotype and several sleep disorders, including difficulty in falling asleep, waking up too early, waking up too tired, waking up at night, and intake of sleep medicine. However, the collection of information on sleep history from age of 20 to the date of reference was based on the self-reported data, making it prone to recall bias that may have affected calculated averages over a lifetime. Kripke et al. showed that self-reported sleep hours tend to be greater than objectively measured hours, which can lead to misclassification [55]. In addition, while we constructed the SDI, all five sleep related difficulties were given the same weightage, which could have introduced bias. Chronotype was assessed subjectively and not by validated testing tools (e.g., the Munich ChronoType Questionnaire or Morningness-Eveningness Questionnaire). Unfortunately, data on type of shifts (fixed vs. rotating), direction and rate of shift rotation, rest periods after shift work, social jet lag, sleep efficacy, or light-at-night exposure during bedtime were not available. Lastly, we were not able to take into account other sleep disorders (snoring, sleep apnea, etc.), or the sleep environment (noise, sleep location, etc.), or the consequences of sleep disorders (depressive symptoms, daytime sleepiness, etc.).

## 5. Conclusions

Our findings suggest that long sleep duration is associated with an increased risk of lung cancer in women. We did not find an overall association between night shift work and lung cancer, but stronger associations were observed in women who worked longer at night and had short sleep durations or high sleep disorders. Further epidemiological studies are required to better understand the potential interaction between sleep traits and night shift work in lung cancer etiology.

## Figures and Tables

**Table 1:** Characteristics of cases and controls.

	Cases (n = 716)		Controls (n = 758)		*p* Value *
	n	%	n	%	
Age (years)					
<50	95	13	86	11	
50–59	196	27	175	23	0.10
60–69	309	43	353	47	
≥70	116	16	144	19	
Area of residence					
75 (Paris)	289	40	316	42	
77 (Seine et Marne)	27	4	22	3	
78 (Yvelines)	32	4	30	4	
91 (Essonne)	24	3	24	3	0.80
92 (Hauts de Seine)	116	16	124	16	
93 (Seine-St Denis)	84	12	73	10	
94 (Val-de-Marne)	130	18	156	21	
95 (Val-d’Oise)	14	2	13	2	
Marital status					
Married/Living with a partner	363	51	323	43	0.01
Single	126	18	168	22	
Separated/Divorced/ Widowed	226	32	267	35	
Socio-professional categories					
Executives and higher intellectual professions	151	21	121	16	<0.001
Intermediate occupations	232	33	278	37	
Employees	264	37	310	41	
Others	58	8	39	5	
Smoking status					
Never smokers	142	20	341	45	<0.0001
Former smokers	201	28	246	33	
Current smokers	373	52	170	23	
Comprehensive Smoking Index					
Never smokers	142	20	341	45	<0.0001
Q1: <0.69	67	9	181	24	
Q2: 0.69–1.45	130	18	116	15	
Q3: 1.46–1.96	173	24	74	10	
Q4: ≥1.96	204	29	44	6	
BMI 2 years before the interview (kg/m^2^)					
Underweight <18.5	62	9	37	5	<0.0001
Normal weight 18.5–24.9	402	56	351	46	
Overweight 25–29.9	161	23	207	27	
Obesity ≥30	90	13	162	21	
Histological type of cancer					
Non-small cell carcinoma					
Adenocarcinoma	514	72			
Squamous cell carcinoma	67	9			
Large cell carcinoma	28	4			
Other types of non-small carcinoma	19	3			
Small cell carcinoma	88	12			

Abbreviation: BMI: Body Mass Index; SD: standard deviation. * *p*-value of the Chi square test.

**Table 2:** Association between sleep traits, chronotype and night work characteristics and lung cancer.

	Cases (n = 716)		Controls (n = 758)		OR *	(95% CI)
	n	%	n	%		
Sleep duration (hours)						
<7	242	34	254	34	1.16	[0.86–1.56]
<6	85	12	85	11	1.22	[0.81–1.85]
6–6.9	157	22	169	22	1.13	[0.81–1.57]
7–7.9	212	30	260	34	1	ref
≥8	260	36	241	32	1.39	[1.04–1.86]
8–8.9	194	27	184	24	1.36	[0.99–1.85]
≥9	66	9	57	8	1.51	[0.95–2.40]
Sleep Disorder Index						
0–1 (low)	304	43	328	43	1	ref
2–3 (medium)	242	34	251	33	0.92	[0.70–1.22]
4–5 (high)	170	24	179	24	0.95	[0.70–1.29]
Chronotype						
Rather in morning	317	44	344	45	1.38	[0.96–1.99]
Neutral type	85	12	113	15	1	ref
Rather in evening	314	44	301	40	1.19	[0.82–1.73]
Night work						
Never	630	88	670	88	1	ref
Ever	85	12	88	12	1.06	[0.74–1.53]
Duration of night work (years)						
Never	630	88	670	88	1	ref
<5	36	5	37	5	1.09	[0.64–1.86]
<3	17	2	21	3	0.89	[0.42–1.88]
3–4.9	19	3	16	2	1.32	[0.63–2.79]
≥5	49	7	51	7	1.04	[0.65–1.67]
5–9.9	23	3	18	2	1.13	[0.55–2.33]
≥10	26	4	33	4	0.99	[0.53–1.80]
Maximum frequency of night work						
Never night work	630	88	670	88	1	ref
Occasionally	30	4	36	5	1.15	[0.65–2.01]
Often	32	5	22	3	1.08	[0.58–2.01]
Always	23	3	30	4	0.95	[0.50–1.79]

Abbreviations: CI: Confidence interval, OR: Odds ratio, SDI: Sleep Disorder Index. * Models adjusted for age, area of residence, marital status, socio-professional category, CSI and BMI 2 years before the interview.

**Table 3:** Association between sleep traits, chronotype and night work and lung cancer according to smoking status.

	Never Smokers						Former Smokers						Current Smokers						
	Cases (n = 142)		Controls (n = 341)		OR * (95% CI)		Controls (n = 341)		Controls (n = 246)		OR * (95% CI)		Cases (n = 373)		Controls (n = 170)		OR * (95% CI)		*p*
	n	%	n	%			n	%	n	%			n	%	n	%			Interaction
Sleep duration (hours)																			
<7	42	30	106	31	1.24	[0.72–2.14]	59	29	91	37	0.88	[0.51–1.54]	141	38	57	34	1.34	[0.81–2.21]	0.549
7–7.9	43	31	119	35	1	ref	59	29	77	31	1	ref	110	30	63	38	1	ref	
≥8	56	40	115	34	1.40	[0.84–2.32]	83	41	78	32	1.33	[0.78–2.27]	121	33	48	29	1.48	[0.87–2.52]	
Sleep Disorder Index																			
0–1 (low)	72	51	151	44	1	ref	97	48	102	42	1	ref	135	36	74	44	1	ref	0.031
2–3 (medium)	43	30	106	31	0.80	[0.49–1.31]	62	31	83	34	0.77	[0.46–1.28]	137	37	62	37	1.25	[0.78–2.01]	
4–5 (high)	27	19	84	25	0.67	[0.38–1.16]	42	21	61	25	0.61	[0.34–1.07]	101	27	34	20	1.88	[1.09–3.26]	
Chronotype																			
Morning type	72	51	173	51	1.43	[0.72–2.83]	86	43	114	46	1.01	[0.52–1.97]	159	43	57	34	1.80	[0.95–3.44]	0.380
Neutral type	15	11	49	14	1	ref	29	14	35	14	1	ref	41	11	28	17	1	ref	
Evening type	55	39	119	35	1.66	[0.82–3.35]	86	43	97	39	0.87	[0.44–1.72]	173	46	85	50	1.22	[0.65–2.29]	
Night Work																			
Never	133	94	300	88	1	ref	173	87	218	89	1	ref	324	87	151	89	1	ref	0.107
Ever	9	6	41	12	0.57	[0.26–1.24]	27	14	28	11	1.53	[0.79–2.96]	49	13	19	11	1.35	[0.72–2.52]	
Duration of night work (years)																			
Never	133	94	300	88	1	ref	173	87	218	89	1	ref	324	87	151	89	1	ref	0.140
<5	2	1	17	5	0.27	[0.06–1.23]	13	7	14	6	1.73	[0.69–4.33]	21	6	6	4	2.13	[0.76–5.97]	
≥5	7	5	24	7	0.83	[0.33–2.09]	14	7	14	6	1.36	[0.56–3.33]	28	8	13	8	1.01	[0.47–2.19]	

Abbreviations: CI: Confidence interval, OR: Odds ratio. * Models adjusted for age, area of residence, marital status, socio-professional category, BMI 2 years before the interview and CSI (only for current and former smokers).

**Table 4:** Association between sleep traits, chronotype and lung cancer according to night work.

	No Night Work						Ever Night Work <5 Years						Ever Night Work ≥5 Years						*p* Interaction
	Cases (n = 630)		Controls (n = 670)		OR * (95% CI)		Cases (n = 36)		Controls (n = 37)		OR * (95% CI)		Cases (n = 49)		Controls (n = 51)		OR * (95% CI)		
	n	%	n	%			n	%	n	%			n	%	n	%			
Sleep duration (hours)																			
<7	202	32	225	34	1.08	[0.78–1.48]	14	39	14	38	0.62	[0.12–3.21]	25	51	15	29	3.23	[1.05–9.90]	0.251
7–7.9	190	30	229	34	1	ref	11	31	12	32	1	ref	11	22	19	37	1	ref	
≥8	236	38	213	32	1.39	[1.02–1.89]	11	31	11	30	1.00	[0.20–5.02]	13	27	17	33	1.78	[0.53–5.99]	
Sleep Disorder Index																			
0–1 (low)	272	43	289	43	1	ref	15	42	15	41	1	ref	16	33	24	47	1	ref	0.140
2–3 (medium)	218	35	226	34	0.95	[0.71–1.27]	10	28	8	22	0.97	[0.19–4.83]	14	29	17	33	0.76	[0.24–2.45]	
4–5 (high)	140	22	155	23	0.90	[0.65–1.25]	11	31	14	38	0.55	[0.12–2.45]	19	39	10	20	2.75	[0.84–9.02]	
Chronotype																			
Morning type	284	45	316	47	1.37	[0.94–2.02]	13	36	12	32	2.40	[0.34–16.8]	20	41	16	31	0.99	[0.18–5.44]	0.805
Neutral type	77	12	101	15	1	ref	3	8	7	19	1	ref	5	10	5	10	1	ref	
Evening type	269	43	253	38	1.21	[0.82–1.79]	20	56	18	49	1.82	[0.23–14.1]	24	49	30	59	0.51	[0.10–2.75]	

Abbreviations: CI: Confidence interval, OR: Odds ratio. * Models adjusted for age, area of residence, marital status, socio-professional category, CSI and BMI 2 years before the interview.

**Table 5:** Association between sleep traits, chronotype and night work and histological subtypes of lung cancer.

	Controls n = 758		Adenocarcinoma (1) n = 514				Squamous Cell Carcinoma (2) n = 67				Small Cell Carcinoma (3) n = 88				*p* Homogeneity Test		
	n	%	n	%	OR * (95% CI)		n	%	OR * (95%CI)		n	%	OR * (95%CI)		(1)/(2)	(1)/(3)	(2/3)
Sleep duration (hours)																	
<7	254	34	177	35	1.27	[0.93–1.74]	16	24	0.50	[0.25–1.02]	36	41	1.03	[0.57–1.88]	0.010	0.489	0.092
7–7.9	260	34	145	28	1	ref	26	39	1	ref	28	32	1	ref			
>8	241	32	190	37	1.43	[1.05–1.94]	25	37	1.04	[0.55–1.96]	24	27	0.95	[0.51–1.79]	0.324	0.201	0.835
Sleep Disorder Index																	
0–1 (low)	328	43	230	45	1	ref	27	40	1	ref	30	34	1	ref			
2–3 (medium)	251	33	162	32	0.87	[0.65–1.16]	29	43	1.19	[0.65–2.16]	34	39	1.16	[0.65–2.06]	0.300	0.317	0.946
4–5 (high)	179	24	122	24	0.97	[0.71–1.34]	11	16	0.60	[0.28–1.30]	24	27	1.21	[0.65–2.29]	0.216	0.481	0.127
Chronotype																	
Morning type	344	45	236	46	1.31	[0.89–1.92]	20	30	1.11	[0.45–2.79]	35	40	2.64	[1.01–6.91]	0.730	0.147	0.167
Neutral type	113	15	64	13	1	ref	8	12	1	ref	6	7	1	ref			
Evening type	301	40	214	42	1.13	[0.76–1.67]	39	58	1.67	[0.71–3.92]	47	53	2.38	[0.93–6.09]	0.368	0.116	0.551
Night work																	
Never	670	88	450	88	1	ref	60	90	1	ref	79	90	1	ref			
Ever	88	12	63	12	1.10	[0.75–1.61]	7	10	0.99	[0.42–2.36]	9	10	0.86	[0.39–1.91]	0.814	0.542	0.799
Duration of night work (years)																	
Never	670	88	450	88	1	ref	60	90	1	ref	79	90	1	ref			
<5	37	5	25	5	1.04	[0.59–1.84]	4	6	1.60	[0.51–5.02]	5	6	1.42	[0.49–4.15]	0.452	0.556	0.867
≥5	51	7	38	7	1.14	[0.70–1.87]	3	5	0.64	[0.18–2.26]	4	5	0.57	[0.18–1.75]	0.362	0.210	0.876

Abbreviations: CI: Confidence Interval, OR: Odds ratio, SDI: Sleep Disorder Index. * Models adjusted for age, area of residence, marital status, socio-professional category, CSI and BMI 2 years before the interview.

## Data Availability

The data that support the findings of this study are available from the corresponding author upon reasonable request.

## Supplementary Materials

The following supporting information can be downloaded at: https://www.mdpi.com/article/10.3390/ijerph192316246/s1, Table S1: Association between sleep traits, chronotype and lung cancer according to night work (never, <10 years, ≥10 years); Table S2: Association between sleep traits, night work and lung cancer according to chronotype.

## Appendix A

**The WELCA study group includes the investigators:** Angelergues Antoine (hôpital St Louis), Arame Alex (Hôpital Européen Georges Pompidou), Arrondeau Jennifer (hôpital Cochin), Badia Alain (Hôpital Européen Georges Pompidou), Baud Marie-Henriette (hôpital Tenon), Bergeron Anne (hôpital St Louis), Boffa Claire (hôpital Cochin), Boudabous Hanene (hôpital Avicenne), Bousquet Guilhem (hôpital Avicenne), Brosseau Solenn (hôpital Bichat), Burgel Pierre-Régis (hôpital Cochin), Bylicki Olivier (hôpital Percy), Cadranel Jacques (hôpital Tenon), Camuset Juliette (hôpital Tenon), Canellas Anthony (hôpital Tenon), Carlier Nicolas (hôpital Cochin), Chaabane Nouha (hôpital Tenon), Chapron Jeanne (hôpital Cochin), Chinet Thierry (hôpital Ambroise Paré), Chohra Abdelaziz (hôpital Cochin), Chouahnia Abdelkader (hôpital Avicenne), Chouaid Christos (Centre hospitalier inter-communal de Créteil), Combe Pierre (Hôpital Européen Georges Pompidou), Crequit Perrine (hôpital Tenon), Crestani Bruno (hôpital Bichat), De Torcy Marie (hôpital St Joseph), Doubre Hélène (hôpital Foch), Doucet Ludovic (hôpital St Louis), Duchemann Boris (hôpital Avicenne), Dumenil Coraline (hôpital Ambroise Paré), Dumoulin Jennifer (hôpital Ambroise Paré), Dusser Daniel (hôpital Cochin), Epaud Christelle (hôpital Tenon), Fabre Elisabeth (Hôpital Européen Georges Pompidou), Fallet Vincent (hôpitaux St Joseph et Tenon), Febvre Michel (hôpital Tenon), Fraboulet Séverine (hôpital Foch), François Thierry (hôpital Tenon), Friard Sylvie (hôpital Foch), Gazaniol Claire (hôpital St Joseph), Giol Mihaela (hôpital Tenon), Giraud Frédérique (hôpital Cochin), Giraud Philippe (Hôpital Européen Georges Pompidou), Giraud Violaine (hôpital Ambroise Paré), Giroux-Leprieur Etienne (hôpital Ambroise Paré), Gounant Valérie (hôpitaux Tenon et Bichat), Hajouji-Idrissi Linda (hôpital Foch), Hamard Cécile (hôpital Cochin), Honoré Isabelle (hôpital Cochin), Issoufaly Tesnime (hôpital Tenon), Jabot Laurence (Centre hospitalier inter-communal de Créteil), Jagot Jean-Luc (hôpital St Joseph), Jouinot Anne (hôpital Cochin), Jouveshomme Stéphane (hôpital St Joseph), Kerleveo Adeline (hôpital Avicenne), Khalife Hocquemiller Thérèse (hôpital Tenon), Labrune Sylvie (hôpital Ambroise Paré), Lafay Michel (hôpital St Joseph), Lavolé Armelle (hôpital Tenon), Le Floch Hervé (hôpital Percy), Le Maignan Christine (hôpital St Louis), Le Pimpec-Barthes Françoise (Hôpital Européen Georges Pompidou), Legras Antoine (Hôpital Européen Georges Pompidou), Lurie Alain (hôpital Cochin), Margery Jacques (hôpital Percy), Massiani Marie-Ange (hôpital Foch), Métivier Anne-Cécile (hôpitaux Foch et Bichat), Monnet Claire-Marie (hôpital St Joseph), Monnet Isabelle (Centre hospitalier inter-communal de Créteil), Mourtada Leila (hôpital Tenon), Naccache Jean Marc (hôpital Tenon), Naltet Charles (hôpital Bichat), Pailler Marie-Christine (hôpital Avicenne), Parent Florence (hôpital Bicêtre), Pastre Jean (Hôpital Européen Georges Pompidou), Pécuchet Nicolas (Hôpital Européen Georges Pompidou), Pouessel Damien (hôpital St Louis), Pricopi Ciprian (Hôpital Européen Georges Pompidou), Prosper Michel (hôpital St Joseph), Rivaud Elisabeth (hôpital Foch), Rivière Frédéric (hôpital Percy), Rosencher Lise (hôpital Tenon), Rousseau Gaëlle (Centre hospitalier inter-communal de Créteil), Rozensztajn Nathalie (hôpital Tenon), Ruppert Anne-Marie (hôpital Tenon), Sahut d’Izarn Marine (hôpital Ambroise Paré), Salles Yvan (hôpital Percy), Salmeron Sergio (hôpital St Joseph), Thibault Constance (Hôpital Européen Georges Pompidou), Vaylet Fabien (hôpital Percy), Vieira Thibault (hôpital Tenon), Vinas Florent (Centre hospitalier inter-communal de Créteil), Zelek Laurent (hôpital Avicenne).
