# Supplementary material for: Sleep Traits, Night Shift Work and Lung Cancer Risk among Women: Results from a Population-Based Case-Control Study in France (The WELCA Study)
Source: Int J Environ Res Public Health. 2022 Dec 4;19(23):16246. doi: 10.3390/ijerph192316246 (PMC9740028; doi:10.3390/ijerph192316246)
Supplement: Supplementary file 1 [file ijerph-19-16246-s001.zip › ijerph-2023991-supplementary.pdf]

Table S1. Association between sleep traits, chronotype and lung cancer according to night work duration (never, <10 years, ≥10 years).

	No night work						Ever night work <10 years						Ever night work ≥10 years					
	Cases (n=630)		Controls (n=670)		OR* (95% CI)		Cases (n=59)		Controls (n=55)		OR* (95% CI)		Cases (n=26)		Controls (n=33)		OR* (95% CI)	
	n	%	n	%			n	%	n	%			n	%	n	%		
**Sleep duration (hours)**																		
<7	202	32	225	34	1.08	[0.78-1.48]	26	44	19	35	1.02	[0.33-3.19]	13	50	10	30	3.62	[0.69-18.9]
7-7.9	190	30	229	34	1	ref	16	27	18	33	1	ref	6	23	13	39	1	ref
≥8	236	38	213	32	1.39	[1.02-1.89]	17	29	18	33	1.08	[0.32-3.63]	7	27	10	30	1.28	[0.21-7.81]
**Sleep Disorder Index**																		
0-1 (low)	272	43	289	43	1	ref	23	39	23	42	1	ref	8	31	16	49	1	ref
2-3 (medium)	218	35	226	34	0.95	[0.71-1.27]	17	29	15	27	0.60	[0.18-2.01]	7	27	10	30	0.86	[0.15-4.88]
4-5 (high)	140	22	155	23	0.90	[0.65-1.25]	19	32	17	31	0.77	[0.24-2.50]	11	42	7	21	5.88	[1.15-30.1]
**Chronotype**																		
Morning type	284	45	316	47	1.37	[0.94-2.02]	20	34	16	29	1.87	[0.41-8.56]	13	50	12	36	0.50	[0.06-4.52]
Neutral type	77	12	101	15	1	ref	5	9	9	16	1	ref	3	12	3	9	1	ref
Evening type	269	43	253	38	1.21	[0.82-1.79]	34	58	30	55	1.83	[0.42-7.99]	10	39	18	55	0.19	[0.02-1.85]

Abbreviations: CI: Confidence interval, OR: Odds ratio

* Models adjusted for age, area of residence, marital status, socio-professional category, CSI and BMI 2 years before the interview

Table S2. Association between sleep traits, night work and lung cancer according to chronotype.

	Morning type					Neutral type					Evening type					
	Cases (n= 373)		Controls (n= 170)		OR* (95% CI)	Cases (n= 201)		Controls (n= 246)		OR* (95% CI)	Cases (n= 142)		Controls (n= 341)		OR* (95% CI)	p interaction
	n	%	n	%		n	%	n	%		n	%	n	%		
**Sleep duration (hours)**																
<7	109	34	110	32	1.29 [0.82-2.01]	32	39	52	46	0.75 [0.32-1.78]	101	32	92	31	1.23 [0.77-1.97]	*0.921*
7-7.9	96	30	122	36	1 ref	23	28	34	30	1 ref	93	30	104	35	1 ref	
≥8	112	35	111	32	1.42 [0.92-2.19]	28	34	27	24	1.54 [0.61-3.90]	120	38	103	34	1.41 [0.90-2.23]	
**Sleep Disorder Index**																
0-1 (low)	145	46	150	44	1 ref	33	39	43	38	1 ref	126	40	135	45	1 ref	*0.741*
2-3 (medium)	103	33	121	35	0.75 [0.50-1.12]	34	40	39	35	1.53 [0.67-3.49]	105	33	91	30	1.05 [0.68-1.64]	
4-5 (high)	69	22	73	21	0.87 [0.55-1.39]	18	21	31	27	0.97 [0.37-2.53]	83	26	75	25	1.02 [0.63-1.64]	
**Night work**																
Never	284	90	316	92	1 ref	77	91	101	89	1 ref	269	86	253	84	1 ref	*0.883*
Ever	33	10	28	8	1.22 [0.67-2.25]	8	9	12	11	1.31 [0.38-4.57]	44	14	48	16	1.04 [0.61-1.77]	

Abbreviations: CI: Confidence interval, OR: Odds ratio

* Models adjusted for age, area of residence, marital status, socio-professional category, CSI and BMI 2 years before the interview
